# Titanium alloy cannulated screws and biodegradable magnesium alloy bionic cannulated screws for treatment of femoral neck fractures: a finite element analysis

**DOI:** 10.1186/s13018-021-02665-2

**Published:** 2021-08-18

**Authors:** Kai Ding, Weijie Yang, Jian Zhu, Xiaodong Cheng, Haicheng Wang, Du Hao, Song Yinuo, Yanbin Zhu, Yingze Zhang, Wei Chen, Qi Zhang

**Affiliations:** 1 Trauma Emergency Center, Key Laboratory of Biomechanics and Orthopaedic Research Institute of Hebei Province, The Third Hospital of Hebei Medical University, No.139 Ziqiang Road, Shijiazhuang, 050051 Hebei People’s Republic of China; 2 School of Medicine, Nankai University, Tianjin, 300071 People’s Republic of China; 3 The First Affiliated Hospital, and College of Clinical Medicine of Henan University of Science and Technology, Luoyang, 471003 China; 4 Yanjing Medical College, Capital Medical University, Beijing, China; 5 Chinese Academy of Engineering, Beijing, 100088 People’s Republic of China; 6 NHC Key Laboratory of Intelligent Orthopeadic Equipment (The Third Hospital of Hebei Medical University), Shijiazhuang, China

**Keywords:** Femoral neck fracture, Titanium alloy cannulated screws (TTCS), Titanium alloy bionic cannulated screws (TBCS), Degradable magnesium alloy bionic cannulated screws (DMBCS), Finite element analysis, Stress distribution, Biomechanical test

## Abstract

**Background:**

Cannulated screws (CS) are one of the most widely used treatments for femoral neck fracture, however, associated with high rate of complications. In this study, we designed a new type of cannulated screws called degradable magnesium alloy bionic cannulated screws (DMBCS) and our aim was to compare the biomechanical properties of DMBCS, the traditionally used titanium alloy bionic cannulated screws (TBCS) and titanium alloy cannulated screws (TTCS).

**Methods:**

A proximal femur model was established based on CT data of a lower extremity from a voluntary healthy man. Garden type III femoral neck fracture was constructed and fixed with DMBCS, TBCS, and TTCS, respectively. Biomechanical effect which three type of CS models have on femoral neck fracture was evaluated and compared using von Mises stress distribution and displacement.

**Results:**

In the normal model, the maximum stress value of cortical bone and cancellous bone was 76.18 and 6.82 MPa, and the maximum displacement was 5.52 mm. Under 3 different fracture healing status, the stress peak value of the cortical bone and cancellous bone in the DMBCS fixation model was lower than that in the TTCS and TBCS fixation, while the maximum displacement of DMBCS fixation model was slightly higher than that of TTCS and TBCS fixation models. As the fracture heals, stress peak value of the screws and cortical bone of intact models are decreasing, while stress peak value of cancellous bone is increasing initially and then decreasing.

**Conclusions:**

The DMBCS exhibits the superior biomechanical performance than TTCS and TBCS, whose fixation model is closest to the normal model in stress distribution. DMBCS is expected to reduce the rates of post-operative complications with traditional internal fixation and provide practical guidance for the structural design of CS for clinical applications.

## Introduction

Femoral neck fracture is a common fracture in the elderly population, accounting for about 60% of hip fracture [1, 2], and is associated with serious medical and social consequences [3–5]. In addition to total hip replacement, osteosynthesis is a well-established operative method for stabilizing femoral neck fracture and currently the most widely used internal fixation instruments were cannulated screws, sliding hip screws, and proximal femoral locking plates [6]. Especially, cannulated screws (CS) was estimated to be used in 78% of nondisplaced/impacted fractures and 46% of the displaced fractures due to its minimal operative trauma, lower medical care cost, and socioeconomic burden [7, 8]. However, the high rate of complications have compromised the operative outcomes, including but not limited to osteonecrosis of the femoral head (14.3–45%), femoral neck shortening (15.9–30%), and nonunion (8–19%) [9–13].

In theory, bone union should include both cortical bone and trabecular bone union, but the role of the latter has been consistently underestimated or even overlooked. Trabecular bone is the center metabolism of bone tissue and also the center of mechanical transmission, bearing 40% to 70% of the body load in the proximal femur [14]. In one experimental study, researchers demonstrated the entire process of bone fracture that 1.5–6.4% of trabecular bone fracture occurred initially, followed by trabecular bone meshwork fracture and finally the fracture of the cortical bone. Additionally, multiple complications have demonstrated to be related to trabecular bone structure change or suboptimal union, due to decrease of bone mass, premature weight-bearing, or internal fixation malposition [15, 16]. Some scholars also suggested that the difficulty in reconstructing trabecular bone have an important relationship with internal fixation failure [17–19]. Owing to stress shielding and volume occupancy effects, traditional implant blocks the reconstruction of trabecular bone, and the load on the proximal femur cannot be transmitted normally even when the cortical bone fully heals [20].

Given above, our team proposed the concept of bionic fixation and designed the bionic implant with porous structure, which allowed the trabeculae to grow in, known as the bionic implant. It is speculated that reconstruction of the trabeculae with use of such device could improve stress distribution and enhance load conduction. In this study, we use the finite element method to address the biomechanical distinction between this bionic implant and the traditional implants (TBCS and TTCS), with regard to stress distribution and stability.

## Methods and properties

This study has been reviewed and approved by the institutional review board and that it conformed to the provisions of the Declaration of Helsinki. Written informed consent was obtained from the volunteers prior to the study commencement.

### Establishing models of proximal femur

**Fig. 1:**
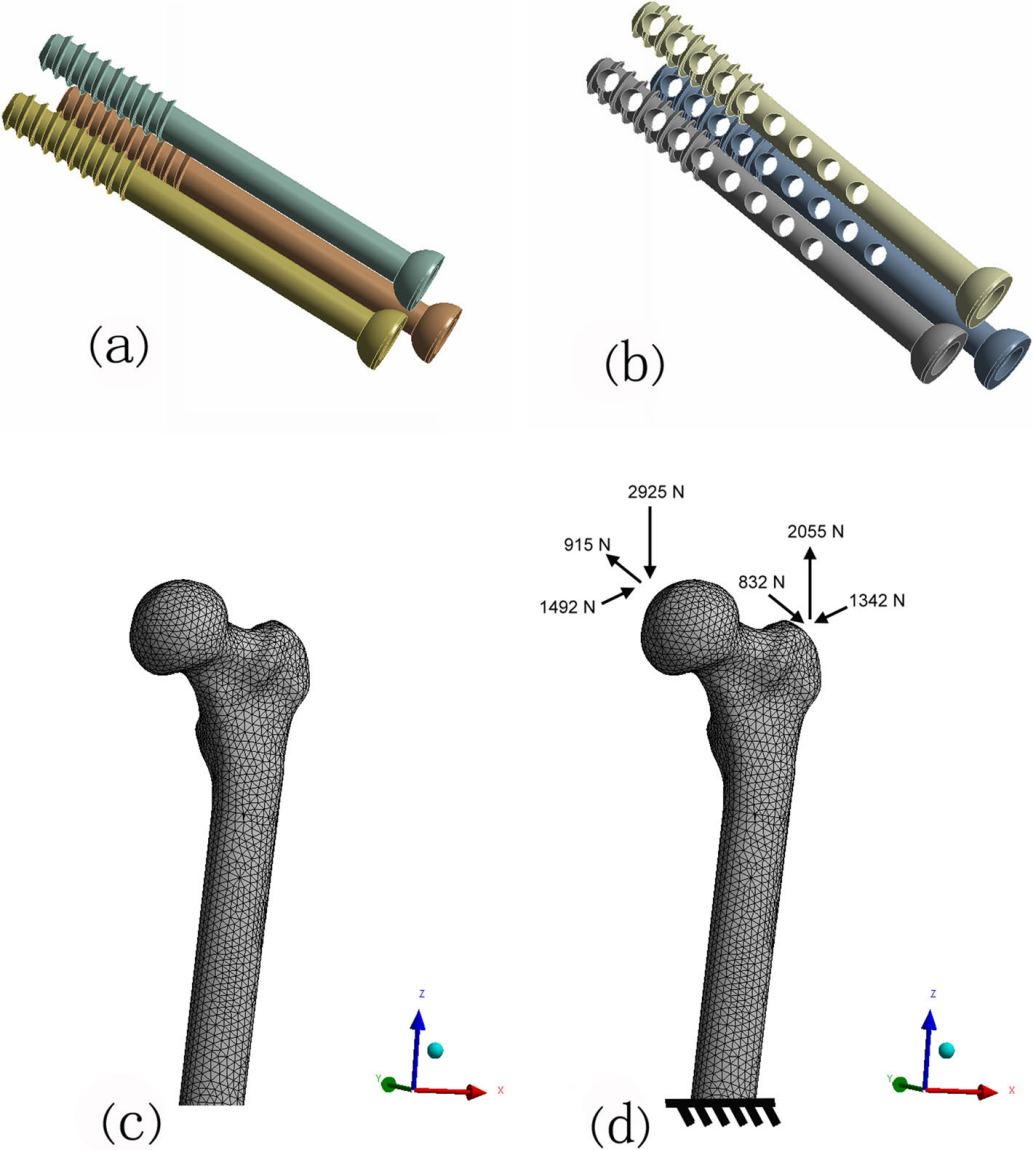
The model of TTCS (**a**), models of DMBCS and TBCS (**b**), the meshed model of proximal femur (**c**), and loading was acted on the model (**d**)

**Fig. 2:**
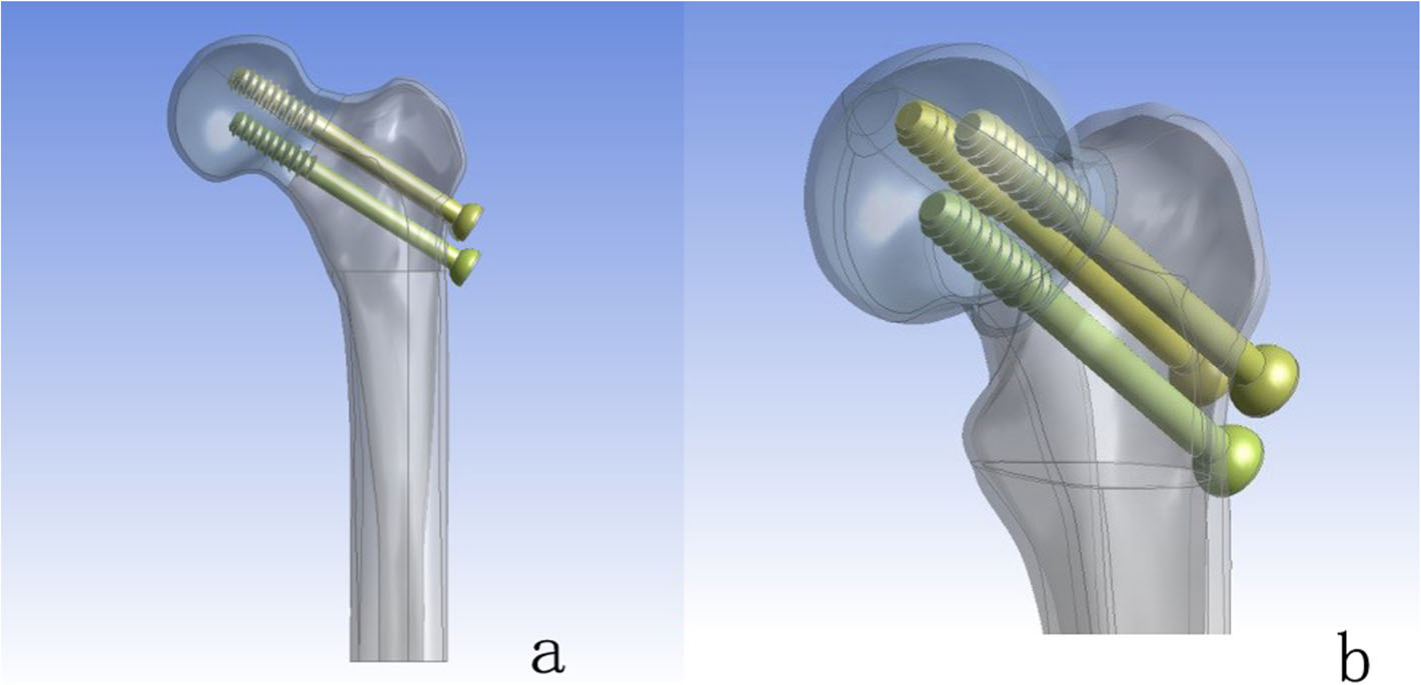
The model of type Garden III femoral neck fracture was established (**a**), placement of CS in proximal femur (**b**)

A healthy volunteer (male, 35 years old, height 170 cm, body weight 75 kg) without a history of lower extremity injury was scanned by computed tomography scanner (SOMATOM Definition AS Siemens, Germany) with a slicing distance of 0.625 mm from hip joint to the knee joint. The three-dimensional femur models were established by these images. The geometry and surface were built and sampled by the Geomagic software. The traditional cannulated screw models were constructed based on their real dimension, and 4-mm diameter holes were created on the bionic implant in NX 9.0 (Fig. 1a, b). A model of Garden type III femoral neck fracture was established by NX 9.0 and fixed with traditional and bionic cannulated screws, respectively (Fig. 2).

### Material properties and boundary conditions

The solid models were imported into Hypermesh13.0 for constructing four-node tetrahedral block-structured meshes of bones and screws. Four-node tetrahedral block-structured meshes of all bones and screws were constructed (Fig. 1c). The models were imported into ABAQUS 6.14. All bone and implant models were assumed to behave with homogeneous, isotropic and linear elastic behavior, and assigned as corresponding material properties according to reported literatures [21] (Table 1).

**Table 1:** Material properties of all models in this study

Model	Materials	Young’s modulus (GPa)	Poisson's ratio
**Cortical bone**	Cortical bone	17	0.3
**Cancellous bone**	Cancellous bone	1.5	0.3
**TTCS**	Ti6Al4V	110	0.316
**TBCS**	Ti6Al4V	110	0.316
**DMBCS**	Mg alloy	45	0.316
**DMBCS (PO 6 months)**	Mg alloy	36	0.316
**DMBCS (PO 12 months)**	Mg alloy	9	0.316

PO represents postoperative

In assembly models, screw thread and cortical screw were bonding with cancellous bone and cortical bone, respectively. The other bone-implant interfaces were set as contact relationship except for thread/bone and cortical screw/bone. The coefficient of friction was set at 0.3 [22].

As the volunteer’s body weight was 75 kg, corresponding to 750 N of gravity, the loading forces on the femur mimicked the loads at the heel strike of normal walking [23]. Figure 1d shows the head load ({*x*, *y*, *z*} = {1492, 915, 2925}) and abductor force ({*x*, *y*, *z*} = {1342, 832, 2055} N) (4.54 and 3.45 times body weight, respectively).

### Evaluation of stress distribution of proximal femoral cancellous bone

The finite element analysis model will simulate 3 different bone healing status: non-healed fracture, partly healed fracture and fully healed fracture. Contact condition of non-healed fracture between the fracture surfaces is simulated as non-sliding but separable, to simulate the situation after the fracture has been repositioned and pressurized. The contact condition of partly healed fracture between the fracture surfaces is set to be combined without slippage or separation for cancellous bone. The contact condition of fully healed fracture is set to be combined without slippage or separation. In addition to the displacement, the analysis records von Mises Stress distribution in bone and screws to evaluate the effect of trabecular bone tissue growth into the fixator on the overall stress distribution.

The von Mises stress on the intact proximal femur was tested to analyze the mesh convergence. The convergence criterion used was a change of < 5%. The cortical bone and cancellous bone of final model had 22,137 elements and 40, 237 elements, respectively.

### Validation of the finite element models

**Fig. 3:**
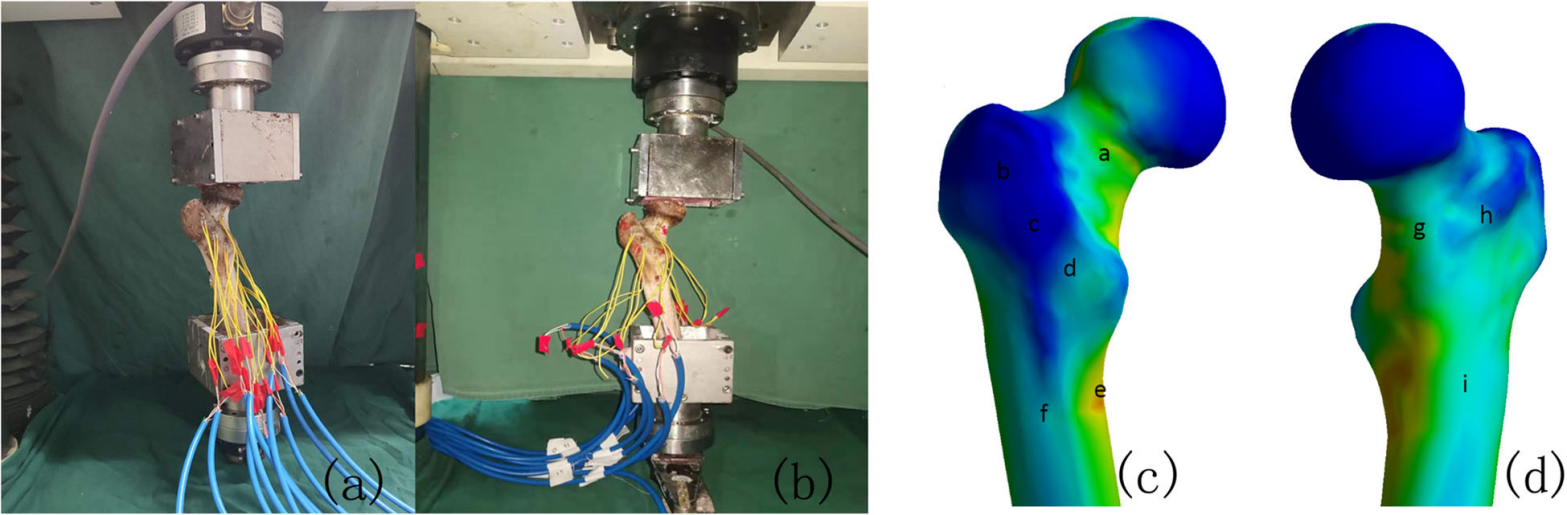
Comparison between the biomechanical test (**a**) and finite element analysis (**b**)

In order to verify the finite element models of proximal femur, a specimen of normal proximal femur was selected for biomechanical test (Fig. 3a). The same load condition and boundary conditions were applied. Further, 750 N load was applied to proximal femur to record strain value of 9 marker points (Fig. 3). The results of comparisons showed that our modelling method is appropriate to be used in the further research, and the difference was not significant (Table 2).

**Table 2:** The strain values of the biomechanical test and finite element analysis (10^−3^)

Maker point	a	b	c	d	e	f	g	h	i
Finite element analysis	4.24	0.02	0.04	1.40	6.53	2.40	5.02	2.52	2.97
Biomechanical test	4.43	0.02	0.04	1.53	6.82	2.75	5.27	2.45	2.67

## Results

### The von Mises stress distribution and displacement of intact bone

**Fig. 4:**
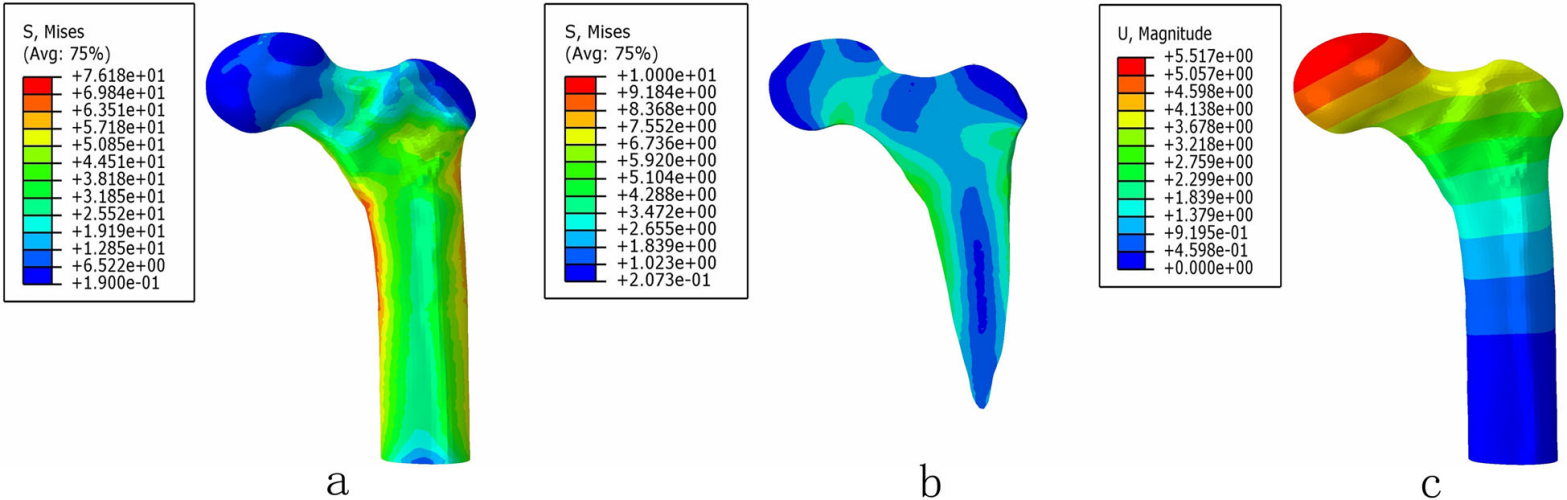
The von Mises stress distribution of cortical bone (**a**) and cancellous bone (**b**); the displacement of the intact models (**c**)

In the normal model, the peak stress values of cortical bone and cancellous bone was 76.18 MPa and 6.82 MPa, respectively. The maximum displacement was 5.52 mm (Fig. 4).

### The von Mises stress distribution, displacement, and shear stress of different CS fixation models in all three settings

**Fig. 5:**
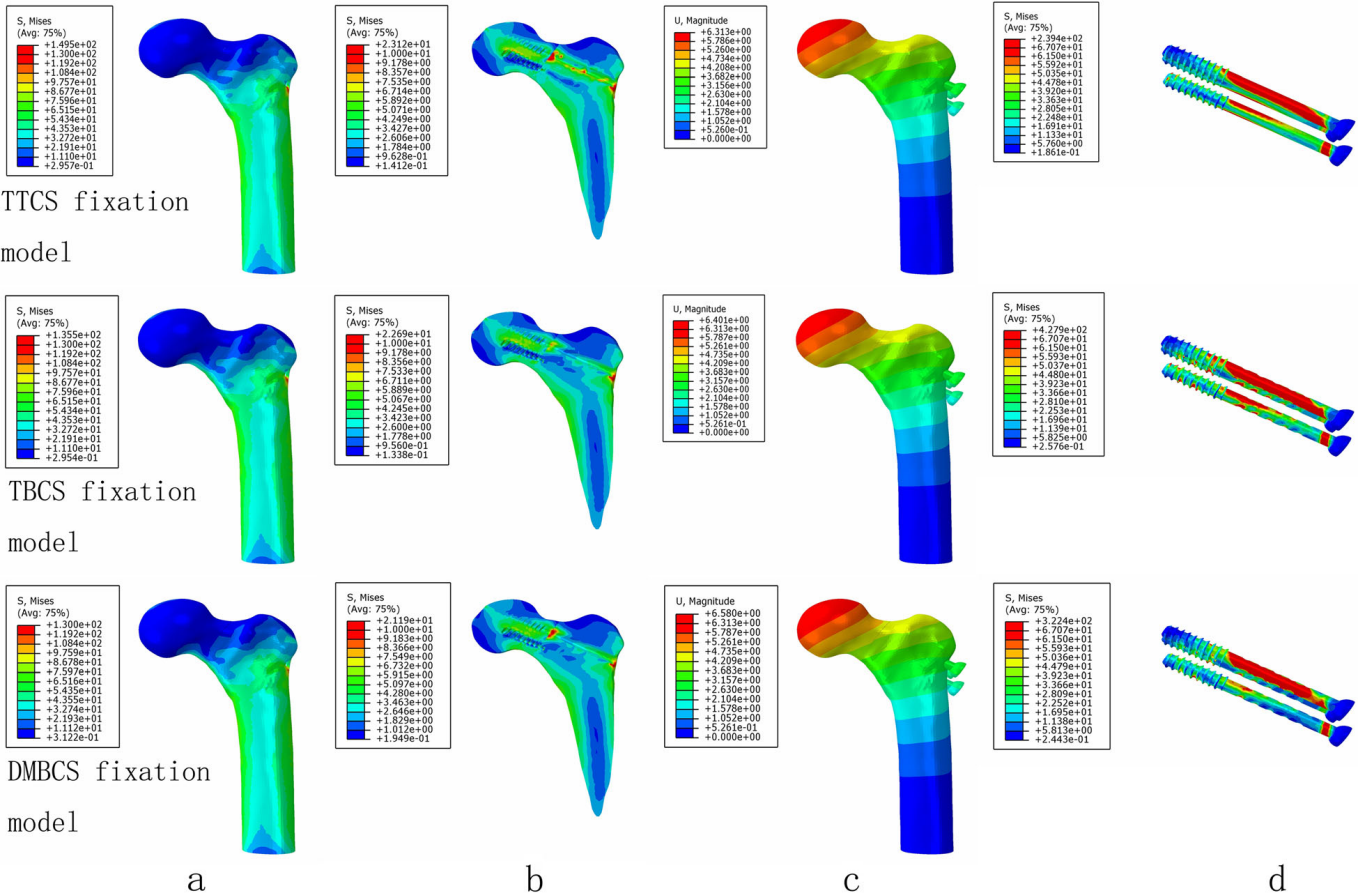
The von Mises stress distribution of cortical bone (**a**), cancellous bone (**b**), and screws (**c**); the displacement of three type of CS fixation models (**d**) at a non-healed fracture

**Fig. 6:**
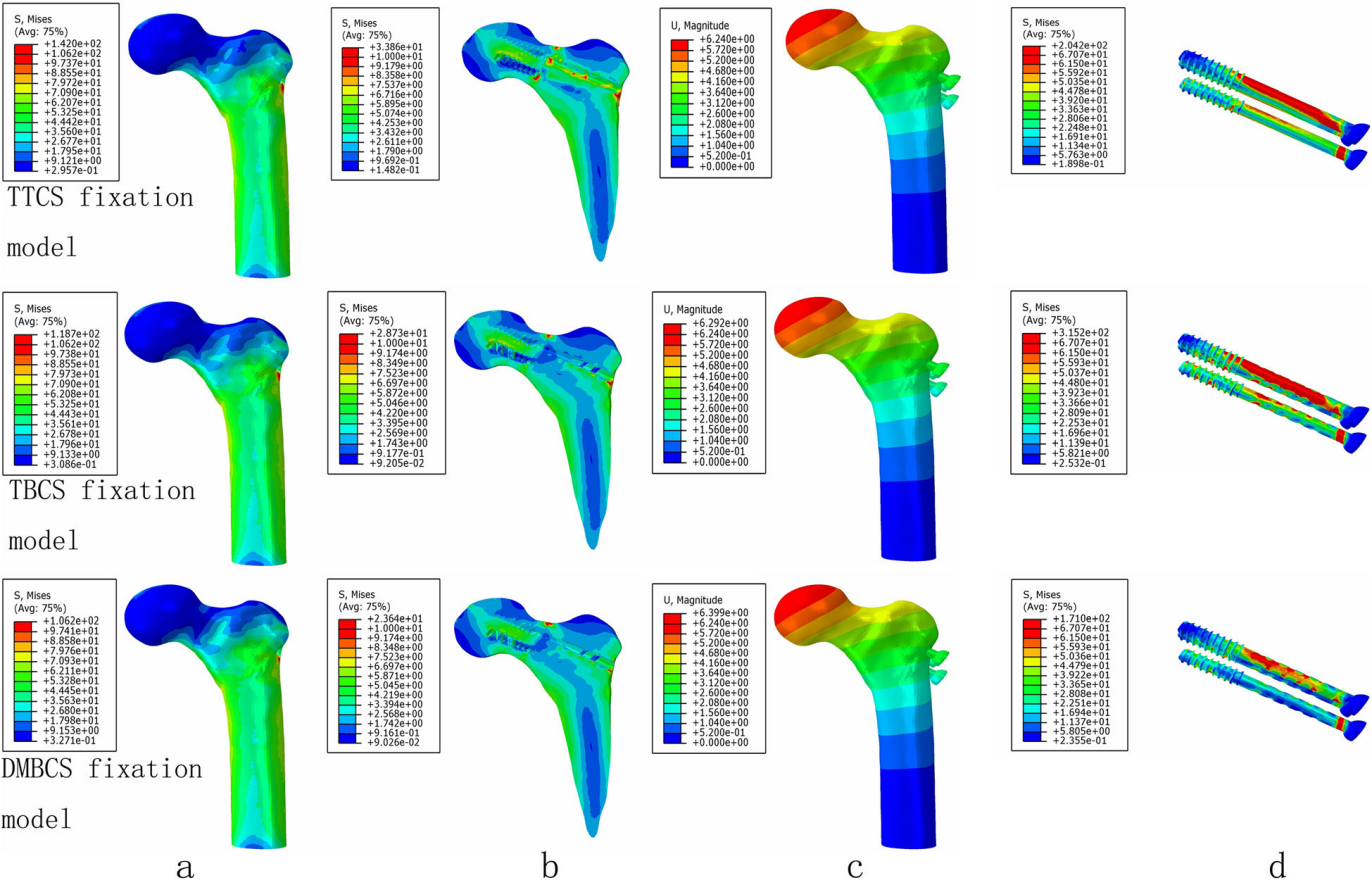
The von Mises stress distribution of cortical bone (**a**), cancellous bone (**b**), and screws (**c**); the displacement of three type of CS fixation models (**d**) at partly healed fracture

**Fig. 7:**
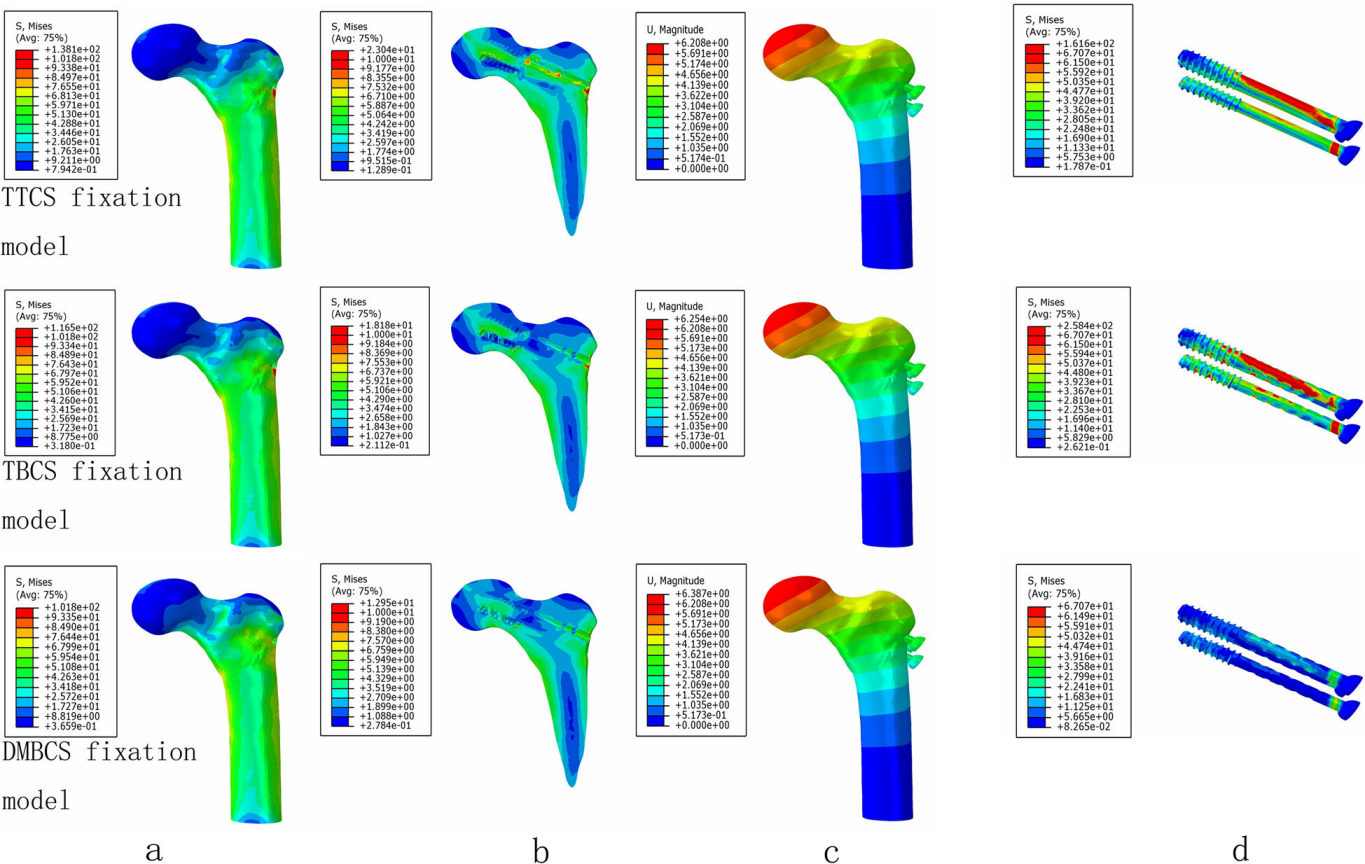
The von Mises stress distribution of cortical bone (**a**), cancellous bone (**b**), and screws (**c**); the displacement of three type of CS fixation models (**d**) at a fully healed fracture

The maximum stress and stress concentration of cortical bone and cancellous bone in the degradable magnesium alloy bionic cannulated screws (DMBCS) fixation model was lower than that in the titanium alloy cannulated screws (TTCS) and titanium alloy bionic cannulated screws (TBCS) fixation models, and the peak bone stress of the three CS fixation models occurred at the holes adjacent to screws. For the screw, the maximum high-stress value of the implant was significantly lower than that in the TTCS and TBCS except a non-healed fracture. High-stress value was located at the middle region of screws among three variations under 3 different bone healing status (Figs. 5, 6, and 7) (Table 3).

**Table 3:** Maximum stress values of screws, cortical bone, and cancellous bone under three healing status (MPa)

Healing status	TTCS			TBCS			DMBCS		
	Screws	Cortical bone	Cancellous bone	Screws	Cortical bone	Cancellous bone	Screws	Cortical bone	Cancellous bone
Non-healed Fracture	239.42	149.54	23.12	427.90	135.50	22.69	322.40	130.00	21.19
Partly healed Fracture	204.23	142.04	33.86	315.16	118.68	28.73	170.97	106.23	23.64
Fully healed Fracture	161.61	138.10	23.04	258.41	116.50	18.18	67.07	101.80	12.95

**Fig. 8:**
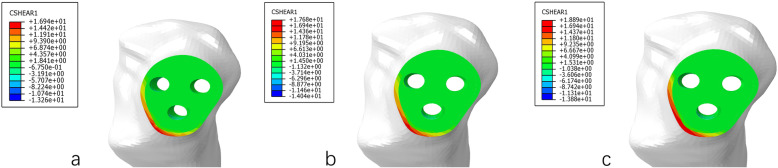
The shear stress distribution of fracture **a** TTCS, **b** TBCS, and **c** DMBCS

DMBCS fixation model was higher than TTCS and TBCS fixation in maximum displacement and shear stress. The maximum shear stress of fracture in three implant groups was 16.94 MPa, 17.68 MPa, and 18.89 MPa at a non-healed fracture (Figs. 5, 6, 7, and 8) (Table 4).

**Table 4:** The maximum displacement of three type of CS under three healing status (mm)

Healing Status	TTCS	TBCS	DMBCS
Non-healed fracture	6.31	6.40	6.58
Partly-healed fracture	6.24	6.29	6.40
Fully healed fracture	6.21	6.25	6.39

## Discussion

Femoral neck fracture is a common fracture in clinical practice, associated with high post-operative complications. To overcome this problem, we designed the new type of CS. In this experiment, the finite element method was used to analyze the stress distribution and stability of DMBCS, TBCS, and TTCS fixation models. Compared with the TTCS and TBCS, the DMBCS has improved stress distribution of the cortical bone and cancellous bone. The result indicates that DMBCS is expected to provide the theory basis for improving clinical efficacy and biomechanical characters of femoral neck fracture.

According to the results, the maximum stress of cortical and cancellous bones in TTCS is 1.15 and 1.09 times greater than that of DMBCS at a non-healed fracture, and 1.35 and 1.78 times greater than that of DMBCS at fully healed fracture. DMBCS has reduced the maximum stress and stress concentration of cortical and cancellous bone in all three settings. The peak value stress of TTCS is 74%, 1.19 times and 2.41 times of DMBCS under three healing status. Except for a non-healed fracture, the stress peak value and stress distribution of DMBCS model are substantially lower than TBCS and TTCS models. DMBCS has improved the stress distribution of fixation models and reduced interference with the surrounding bone.

There are several biomechanical characteristics of DMBCS that explain the biomechanical differences from both traditional and bionic CS. First, the natural elastic modulus of degradable magnesium alloys is low and they are expected to slowly degrade over time. The difference of elastic modulus between cancellous bone and screw is small, and the stress concentration and stress extreme value will also be reduced [23]. This feature is also reflected in the biodegradable internal fixation used for spinal fractures, and the prior study demonstrated the biodegradable material could indeed reduce the stress concentration of the vertebrae during degradation and the stress distribution was closer to the normal model [22]. DMBCS will reduce the disadvantages of stress shielding and the risk of relevant complications [24]. Second, the dynamic change of DMBCS conforms to the healing process of femoral neck fracture. DMBCS could provide rigid support for femoral neck fractures, which contributes to the healing process of bone in the early stage. The load carried by the DMBCS will gradually decrease while the load carried by the femoral neck itself will gradually increase, providing an appropriate stress environment for the growth of the trabecular bone [25]. Last but not least, porous structure was consistent with that of trabecular bone in the proximal femur, which can reduce the volumetric mass effect during fracture healing. It can be theoretically speculated that trabecular bone will gradually replace the screws in DMBCS fixation model, thereby avoiding the surgical removal of screws and hence reducing the need for bone grafting procedures [26]. However, the maximum displacement in TTCS fixation model was 96%, 98%, and 97% of that in DMBCS fixation model under three healing status. The maximum displacement and contact/shear stress of DMBCS fixation model is marginally higher than other type of CS models because porous structure and lower modulus of DMBCS reduce stress shielding and volumetric mass effect. We consider that appropriate stress environment and elastic fixation are beneficial to healing process of fracture. Excessively rigid structures may reduce micromotion at the fracture site, such that it is below the threshold required for callus formation [27, 28].

At present, porous metals have been widely used in joint replacement and bone defects, achieving the better results in terms of prognosis and complications, compared with traditional fixation methods [29–31]. Bionic materials including titanium alloy and tantalum metal demonstrated to effectively promote the ingrowth of trabecular bone [32–34]. However, these metal cannot be completely removed in joint replacement and bone defects [35]. Degradable magnesium alloys can overcome the disadvantage. Moreover, the density and elastic modulus of magnesium alloy are close to bone [36]. In addition to the good biomechanical properties, magnesium is also an essential trace element for the human body. Also, the release of magnesium ions during degradation induces the growth of bone [37]. Degradable magnesium alloy screws have been clinically applied in carpal fractures, thumb valgus, and femoral neck fracture, which show good mechanical properties and biosafety [38–40]. Thus, this is our original intention of designing this device that combines magnesium alloys with porous materials.

The design concept of DMBCS was derived from the special structural and mechanical characteristics of the trabecular bone in the proximal femur. The trabecular bone of the femoral neck is adapted to their role in mechanical conduction [14]. Lotz et al. [41] showed that cancellous bone bears 20%, 4%, 50%, and 70% of the body load in femoral intertrochanteric, basal, middle, and subcapital regions of the femoral neck, respectively. Therefore, decreasing thickness and number of trabecular bones is one of the most important reasons why elderly patients are prone to femoral neck fractures [42]. Nawathe et al. [19] performed 12 femoral specimens to investigate the injury mechanism of femoral neck fractures caused by lateral fall violence and found only about 1.5–6.4% of trabecular bone cracked initially. These studies shown the trabecular bone played an important “initiating role” in the development of femoral neck fractures. Therefore, whether the distribution of trabecular bone after surgery can adapt to the change of external environment and whether the reconstruction of trabecular bone is consistent with the transmission direction of human load will directly affect the prognosis of femoral neck fracture treatment. When designing internal fixation in femoral neck fracture, we need to pay attention to the biphasic reconstruction of cancellous bone and cortical bone during the healing process.

Limitations exist in our study. First, this test neglects that the shape and volume of DMBCS will change during degradation, which may affect the results. Second, the material properties of the cortical bone and cancellous were assumed to be isotropic, linearly elastic, and homogeneous behavior, whereas bones are really composed of anisotropic viscoelastic material.

In summary, DMBCS fixation model is closest to the normal model in the stress distribution, and exhibited better biomechanical performance than the other traditional implants. DMBCS can meet the requirements of dynamic fixation of femoral neck fractures, and reduce the interference on cortical and cancellous bone. DMBCS is a promising internal fixation device for femoral neck fracture.

## Data Availability

Please contact author for data requests.

## Abbreviations

CS: Cannulated screws
TTCS: Titanium alloy cannulated screws
TBCS: Titanium alloy bionic cannulated screws
DMBCS: Degradable magnesium alloy bionic cannulated screws
